# Traditional Chinese martial art Wushu to improve the mental state and physical fitness of students: designing space for optimal practice and training

**DOI:** 10.3389/fpsyg.2025.1581226

**Published:** 2025-06-18

**Authors:** Zhixing Tu, Lei Zhong, Jiahui Xie

**Affiliations:** ^1^Department of Physical Education and Research, Fujian Medical University, Fuzhou, China; ^2^Department of Physical Education Teaching and Research, The Russian University of Sport “GTSOLIFK”, Moscow, Russia; ^3^Department of Physical Education Teaching and Research, Fuzhou University, Fuzhou, China

**Keywords:** acoustic environment, architecture, biomechanics, cultural pedagogy, integration of mind and body, proprioception, ritual elements

## Abstract

**Introduction:**

Traditional Chinese martial arts (Wushu) possess the potential to enhance both the psychological resilience and physical capacities of learners. Their effective implementation necessitates consideration of spatial and architectural features. This study investigates the impact of spatial-architectural determinants on the psychophysiological adaptation of Wushu practitioners within Chinese and European pedagogical systems.

**Methods:**

A mixed-methods approach was employed, combining quantitative biomechanical assessments (*n* = 184 trainees) with qualitative ethnographic observations (24 training settings over 4-week immersion periods) and semi-structured interviews (*n* = 42 instructors). Architectural parameters—including ceiling height, floor elasticity, and acoustic properties—were systematically documented, alongside measurements of biomechanical performance indicators.

**Results and discussion:**

Chinese training environments, characterized by high ceilings (>4 m) and specialized wooden flooring, correlated with superior flexibility (hip flexion: 142° ± 3.6° vs. 130° ± 3.8°; *p* < 0.01) and cardiovascular efficiency (shuttle run: 987 ± 42 m vs. 924 ± 38 m; *p* < 0.01). European facilities, utilizing rigid synthetic materials, demonstrated advantages in explosive strength metrics (vertical jump: 54.3 ± 3.1 cm vs. 50.0 ± 2.9 cm; *p* < 0.05). Ceiling height emerged as the dominant architectural predictor of flexibility parameters (*β* = 0.73, *p* < 0.001); acoustic characteristics significantly influenced exercise execution speed (*β* = −0.68, *p* < 0.001); and spatial volume affected cardiorespiratory adaptation (β = 0.65, *p* < 0.001). Environments incorporating traditional design elements improved attentional resilience by 27% compared to modernized spaces. The application of data-driven spatial design principles—such as the inclusion of ritualized zones, hierarchical spatial organization, and tailored acoustic environments—represents an underexplored domain within martial arts pedagogy with promising implications for educational practice.

## 1 Introduction

Educational paradigms of Wushu reveal significant methodological contradictions. Among the most notable are the lack of robust quantitative frameworks that successfully integrate cognitive and biomechanical synergy, and the insufficient spatial conceptualizations that bridge environmental psychology with kinesthetic learning processes (Wang, 2024). Traditional mechanisms of knowledge transmission increasingly encounter systematic decontextualization within Western pedagogical models, leading to a dilution of cultural and methodological coherence (Zhang et al., 2024).

Modern research trajectories also expose epistemological gaps. There is a notable absence of quantitative data concerning the role of spatial acoustics in the development of proprioceptive skills. Interdisciplinary models that interlink neurocognitive mechanisms with architectural parameters remain underdeveloped, while comparative analyses that account for cultural particularities in methodological efficacy are still scarce. The capacity for psycho-emotional regulation differs substantially between traditional and modernized instructional approaches (Jiang, 2024). Executive functions are rarely synchronized with motor learning protocols, resulting in fragmented cognitive-motor outcomes (Bao et al., 2025). At the same time, psychological readiness demonstrates a measurable correlation with performance outcomes when systematically integrated into training structures (Ying, 2024). Cultural hybridization fosters new configurations of identity. Russian Wushu practitioners, for instance, embody a form of zhongdanren that necessitates the externalization of a “second skin” through the use of uniformed bodily presentation (Santanna and Li, 2024). Coaching support functions as a mediating variable in the relationship between motor skill acquisition and long-term engagement in practice (Jianting, 2025). Explosive strength training protocols effectively reprogram neuromuscular optimization patterns, and the integration of multiple models enables elite athletes to enhance coordination efficiency (Zeng, 2022).

Despite their appeal, contemporary sports education models often disrupt the philosophical coherence of traditional Wushu instruction (Hastie et al., 2021). Within the Chinese context, a growing preference for Olympic-standardized training undermines the authenticity of indigenous cultural frameworks. Failures in implementation often stem from enrichment strategies and de-traditionalizing reforms that introduce conceptual contradictions into the pedagogical process (Han, 2019). Geographic and cultural fragmentation further deepens these divides. African reinterpretations of martial practice often emphasize a composite of combat, dance, and spirituality, while Chinese systems uphold a purist vision of Wushu. These divergences give rise to cultural discontinuities, particularly between codified instructional knowledge and embodied interpretations—often collectively referred to as “Kung Fu” (Rodrigue et al., 2019).

Chinese athletes frequently opt out of formal education to pursue Olympic success, while Western educational frameworks resist integrative compromises. In tightly controlled, centralized training environments, Wushu professionals negotiate highly limited access to educational advancement (Zhang et al., 2019). The transmission of knowledge is becoming increasingly fragmented along methodological lines. While Outcome-Based Education (OBE) platforms facilitate the integration of informational content, they simultaneously contribute to the erosion of Wushu’s philosophical foundations in digital learning contexts. Notably, a 32.82% reduction in Mean Absolute Error (MAE) illustrates technical achievement, yet also signals the risk of cultural dilution in technologically mediated training paradigms (Qian, 2024).

The practice of Wushu has been shown to enhance anaerobic capacity more effectively than alternative martial arts disciplines. Executive functioning correlates with well-designed learning environments, revealing a reduction in perceptual complexity. Metacognitive learning represents an emergent yet underexplored pedagogical trajectory within martial arts education (Bao et al., 2025). The integration of moral and technical components necessitates spatial resolution; achievement motivation flourishes within environments that support holistic practice. Pedagogical improvements are most evident when the physical space aligns with and accommodates a philosophically grounded curriculum (Wang, 2024).

Aesthetic variables exert measurable effects on performance outcomes. Strength, flexibility, and visual expression demonstrate statistically significant regression patterns, indicating that these embodied dimensions are sensitive to environmental and instructional variables (Mei and Yuan, 2024). Errors in performance have been observed in the correlation between two-dimensional movements and three-dimensional joint angles, highlighting the need for architectural compensation to mitigate biomechanical inconsistencies (Chen B., 2024). The efficacy of mental preparation is influenced by the psychoacoustic properties of the surrounding environment, which play a critical role in attentional regulation. Periodized training programs, in turn, demand correspondingly structured physical spaces to optimize progression and recovery (Ying, 2024). The cultivation of hybrid identity is contingent upon bicultural environments, materially expressed through spatial and sensory design (Santanna and Li, 2024).

Modular adaptive architecture emerges as a synthesized framework that preserves traditional values while quantitatively assessing performance-related variables historically considered intangible or immeasurable.

### 1.1 Literature review

Wushu, a martial discipline, forms an ontological structure in which psychophysical regulation, cognitive dexterity, and kinetic sophistication converge, acting as a synthesis of militaristic efficiency and philosophical embodiment (Blumentritt, 2019). The structural logic of its execution – in the context of Zen-infused Shaolin methodology or militarised tactical schemes – requires a balance between controlled aggression and neurophysiological modulation, ensuring precision of movement rather than impulsive application of force (Busol, 2016). The epistemic foundation of Wushu – historically codified in Chinese martial philosophy – articulates a dual imperative: maximising tactical efficiency and cultivating internal balance (Cynarski and Szajna, 2017). A pedagogy of engagement requires biomechanical mastery, the capacity for adaptive recalibration, anticipatory perception, rapid kinesthetic integration, and modulation of affective responses (Cynarski and Johnson, 2020).

**Figure 1:**
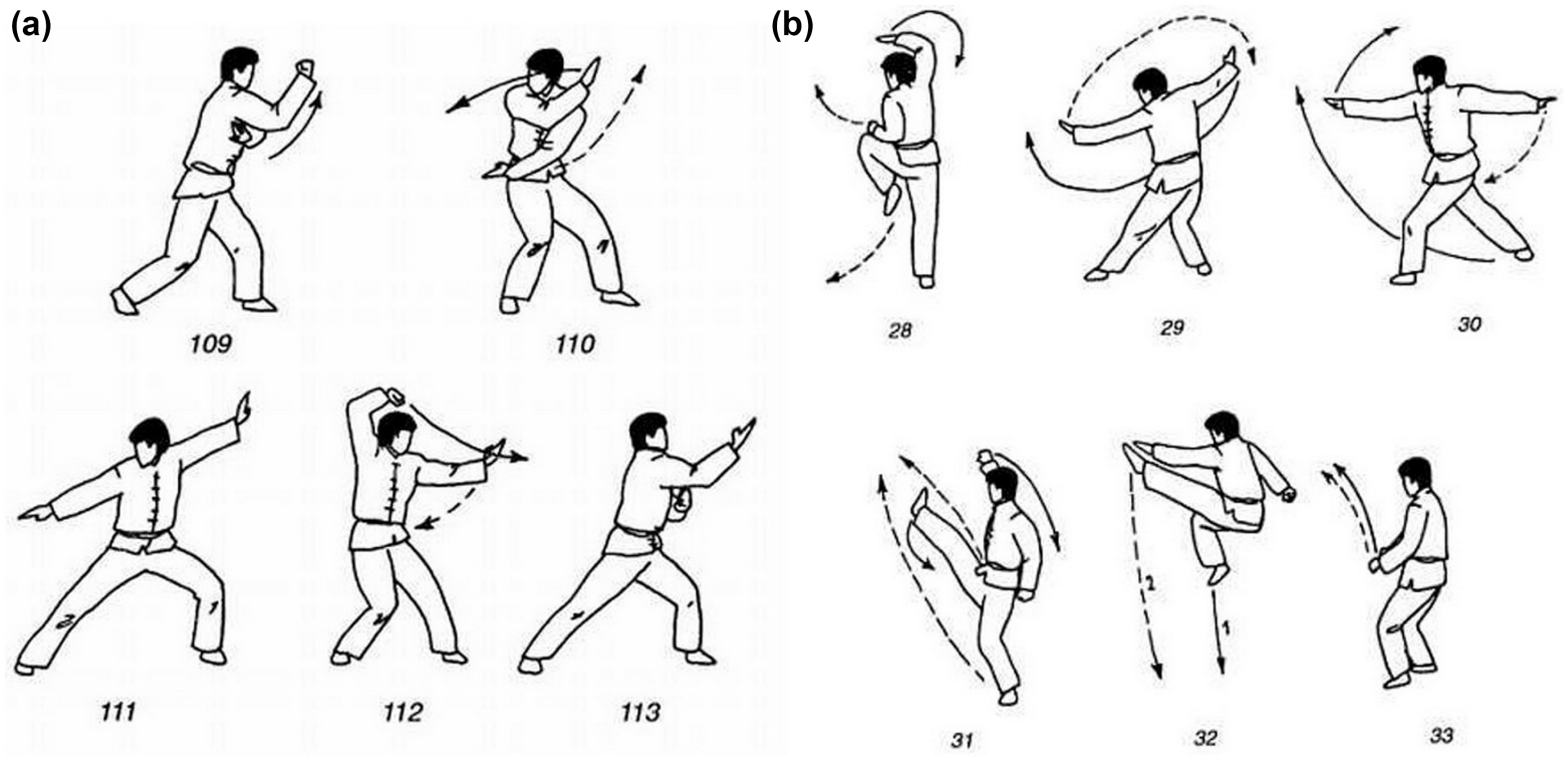
Examples of Wushu barehanded techniques: **(a)** evasive movements; **(b)** attacking Wushu movements.

Wushu’s structural taxonomy, categorised into distinct technical archetypes, reveals its systemic depth: Wushu Taolu, a repertoire emphasising projectile kinetic logic and adaptive strike vectors, conditions the practitioner’s neuro-motor circuitry for multi-vector combat scenarios (Duan, 2017); the kinetic differentiation between Changquan, Taijiquan, Hongquan, and Paoquan – as an evolution of biomechanical sequencing that enables adaptability of skills in changing tactical contexts (Dai, 2016). Competitive codifications – bare-handed performance/quanshu, sequences using weapons/duan qixe, and long-distance fighting methodologies/chang qixe – reflect an institutionalised attempt to distill the pragmatic application of Wushu within a regulated framework (Gajewski and Przewoznik, 2020; Gao and Soonjan, 2024; Gavriely-Nuri, 2017; General Office of the State Council, 2019). The traditional martial sequence – structured as a 16-phase iterative progression – emphasises evasive recalibration, tactical redirection, and strategic phase transitions (Figure 1), reinforcing a fundamental principle: Wushu is a system of embodied cognition in which movement encodes intention and spatial logic dictates strategic viability.

**Figure 2:**
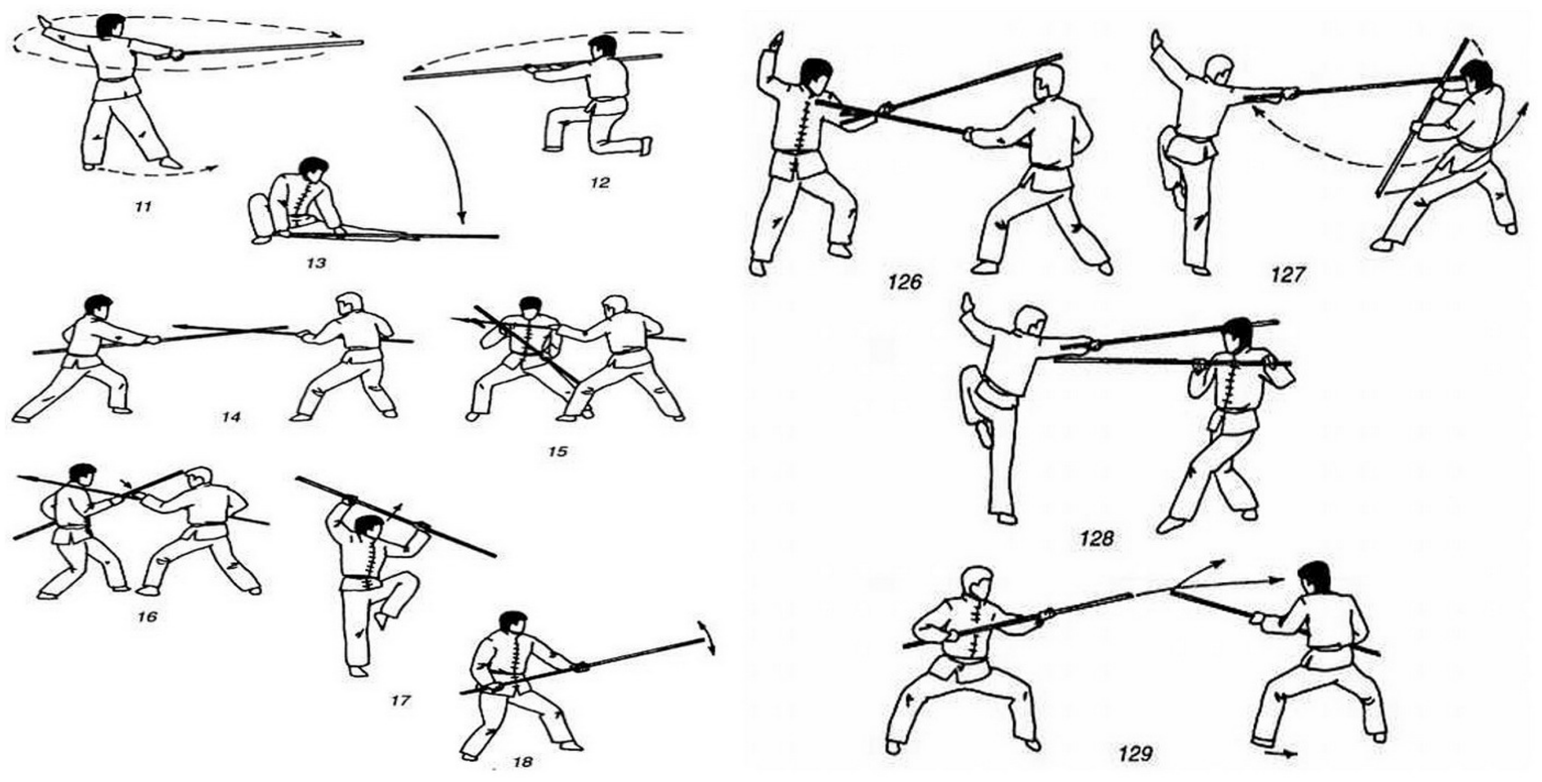
Wushu techniques with combat wooden weapons.

Wushu, the embodied practice of unarmed technique, comprises an extensive arsenal of weapon-based techniques structured within a taxonomic framework that encodes biomechanical efficiency, tactical variation, and neurocognitive adaptability (Cynarski and Johnson, 2020). A codified repertoire – eighteen distinct categories – defines weapon typologies: striking weapons (staff/stick/bludgeon/staff), pointed and stabbing weapons (sword/knife/spear/halberd/hilt/axe/pike), and throwing projectiles (bow/crossbow/stick/slingshot) (Monteiro et al., 2020). Traditional pedagogy integrates these elements through a kinetic sequence – each technique synchronises force transfer, spatial awareness, and anticipatory control – the logic of movement dictates offensive vitality, defensive recalibration (Figure 2).

Adaptive synergy emerges in the pedagogy of ‘Wushu’ (Chen, 2017), a technique imbued with moral foundations that fosters an anthropocentric continuum that goes beyond mere sportsmanship (Park, 2019). The historical tradition emphasises: “a martial art aimed at personal development” (Pawelec, 2020), emphasising the continuous improvement of skills and cultivating an ethos based on respect/collectivism (Kulpinski, 2018). Western interpretations minimise the moral framework (Greitemeyer, 2022), the Chinese approach insists on an ethos that combines discipline with interpersonal synergy (Melguizo-Ibáñez et al., 2023). Daily training begins with a calibrated warm-up, which is a precursor to technical training that reveals “new techniques” related to internal balance (Russ et al., 2017). Mastery, in this context, refers to the smooth interplay of ‘hardness and softness’ (Siswantoyo and Kuswarsantyo, 2017): trainees cultivate psycho-physiological coordination coupled with unwavering attention to self-awareness, to cope with adversity and cultivate psychological resilience (Jarman et al., 2021). Traditional “Wushu” requires progression from basic steps to complex decompositions of movements, forming reflex arcs that hone martial abilities and emotional self-regulation (Qiu and Song, 2017; Yang et al., 2019). Another facet emerges with the integration of advanced technologies – multimedia resources provide enhanced modes of demonstration and analysis (Zilai and Lin, 2018); such cybernetic scaffolding conveys visual parsing of movements, enhancing conceptual clarity and addressing theoretical gaps (Deming, 2018). “Collectivism” proves to be a powerful factor – Western students face friction when hierarchical codes encourage obedience to elders (Cynarski and Johnson, 2020); some researchers find such immersion in group cohesion useful for identity formation and mental balance (Sunardi, 2019). According to experts, the “dark side of sport” is mitigated by rigorous moral training that discourages unwarranted aggression and promotes responsibility (Greitemeyer, 2022). Cross-pollination between cultures leads to “contradictions and misunderstandings”, but mobilising moral precepts shaped by classical Chinese thought can promote self-efficacy, calm, and a sense of belonging (Cynarski, 2019). Discrepancies in judgment persist – over-reliance on digital platforms potentially hinders real-time coach feedback or group momentum, generating motivational pitfalls and technical barriers (Zhang, 2021). The synergy between physical immersion and interactive technologies – provided that specialised equipment needs, barriers to connectivity, and self-discipline are considered – can offer a holistic plan for students seeking “a harmonious lifestyle, constant internal training and struggle” (Siswantoyo and Kuswarsantyo, 2017). The balanced cultivation of body and mind, manifested in the interplay of “technique” and “consciousness”, confirms that despite cultural dissonances, Wushu retains the potential for intellectual hardening and bodily resilience (Partikova, 2019).

The practice of Wushu correlates with increased anaerobic power that exceeds the conventional benchmarks associated with standard physical training (Ma, 2025). Notable evaluative discrepancies emerge between pedagogical perspectives and student learning preferences: while instructors prioritize aesthetic variables, students tend to favor functional kinematics. Outcome assessments reveal systematic misalignments between instructional methods and learners’ cognitive reception (Mei and Yuan, 2024). Functional motor proficiency is a prerequisite for effective performance, and coaching mediation contributes to the development of psychological resilience (Jianting, 2025). Cardiorespiratory adaptation encompasses both physiological and psychological health domains. Eight-week training programs have been shown to improve vital lung capacity alongside indicators of emotional regulation. Measures of flexibility, coordination, and speed-strength concurrently demonstrate psychological enhancement, suggesting that the aerobic/anaerobic dichotomy necessitates tailored programming. Specialized training models outperform generalized strength development when applied to competitive contexts (Li and Siriphan, 2024). Self-determination theory supports the efficacy of personalized instructional approaches within traditional martial arts contexts (Wang and Salleh, 2024).

Safety-oriented learning processes require spatially targeted interventions informed by injury topography (Sun et al., 2022). The monitoring of training-induced fatigue incorporates environmental variables, as athletes’ biochemical indicators fluctuate in response to spatial configurations that influence recovery trajectories. Physical conditions must therefore be integrated with athlete profiles through effective injury prevention methodologies (Ren, 2022). While intelligent resource databases achieve data transmission rates of up to 90%, architectural spatiality remains an overlooked dimension. Personalized training recommendations increasingly draw on behavioral models yet often omit spatial considerations (Wang and Feng, 2023). Socio-economic evaluations underscore the potential for sustainable development via architectural integration, reflected in modest yet measurable indices of foreign direct investment (FDI), service capacity, and urbanization at the 1% level. Difference-in-differences modeling confirms the stimulative impact of martial arts practice on local economies (Liu and Deng, 2024).

Demonstrative learning continues to evolve alongside multimedia technologies while retaining its foundational pedagogical efficacy. Paradigms of psychological intervention have begun to deconstruct the mechanisms underlying athlete stress: entropy-based weighting coefficients are used to model fuzzy judgment matrices, thereby reconstructing coping strategies that improve mood trajectory outcomes (Li, 2020). The methodology of sports education challenges conventional instructional assumptions. Semester-based seasonal formats—structured around six thematic clusters, including teams, competitions, and role assignments—merge into climate-based preferences that surpass traditional enthusiasm metrics (Hastie et al., 2021).

Stratified models of moral education fragment into components of cultural ideology, instructional systems, and philosophical foundations before being reconstructed into systematic theoretical frameworks. Excavating moral values thus requires strategically guided educational interventions. Statistical data mining techniques reveal transformation patterns applicable to pedagogical development (Tao, 2021). Normative standards demand simplification in tandem with coordinated arbitration systems (Han, 2019). Fatigue damage modeling deconstructs both physical and psychological mechanisms during the restructuring of recovery protocols; the specificities of martial arts necessitate targeted restoration strategies (Yu, 2020). Traditional Wushu narratives disaggregate into historical genres—Confucianism with rationality, Daoism with skill, and Buddhism with physical strength. The advancement of traditional martial arts requires a comprehensive understanding of their underlying philosophical interconnections (Wu et al., 2020).

Participation in martial arts is associated with a reduction in symptoms of depression and anxiety under standardized conditions (Moore et al., 2019). The implementation of martial arts training in secondary school settings has been systematically validated for its therapeutic efficacy. Randomized controlled protocols evaluating ten-week interventions show statistically significant psychometric improvements; sessions of 50–60 min, conducted ten times, yield quantifiable enhancements in affective regulation.

The presence of real or imagined opponents facilitates attentional modulation through the symbolic imagery of daggers and axes. These adversaries implicitly redirect practitioners’ focus from the inhuman to the human, and from others to the self (Dai and Lu, 2019). Psychological training complements physical development, particularly in university-based settings, where integrative methods combine mental preparation with biomechanical conditioning. Such training supports optimal personality formation within broader social contexts (Huang, 2019). The expression ratio of PGC1α4 to PGC1α2/3 demonstrates improvements in motor performance, as evidenced by comparative analyses. Traditional hieroglyphic interpretations are increasingly integrated with spatial distribution modeling; Moran’s I correlation (0.172) reveals geographically clustered patterns concentrated in the provinces of Shandong, Henan, and Hebei. Regional cultural ethos significantly influences the spatial distribution of martial arts institutions (Yu et al., 2025).

The optimization of training environments remains a theoretically underexplored domain. Pedagogical frameworks often synthesize constructivist learning theories and self-determination theory while excluding variables related to environmental design. Although student knowledge and value systems demonstrably influence learning outcomes, the role of spatial facilitation continues to receive minimal attention (Wang and Salleh, 2024). Specialized training regimens enhance both aerobic and anaerobic capacities; however, the architectural correlates of performance remain insufficiently studied. Eight-week programs have shown measurable improvements in VO₂max, yet the spatial configurations contributing to these outcomes lack quantitative evaluation. The prevailing emphasis on absolute strength development tends to overlook spatial parameters critical to sparring progression in Wushu (Li and Siriphan, 2024).

Intelligent instructional systems increasingly integrate cultural elements and martial arts practice through posture assessment technologies, yet the broader spatial learning ecosystems remain in need of holistic redesign. Error detection in movement occurs across both 2D and 3D dimensions, but the optimization of the training environment continues to elude algorithmic frameworks. National cultural integration strategies similarly neglect spatial determinants of learning (Chen B., 2024).

Socio-economic analyses frequently employ difference-in-differences modeling, yet architectural value metrics are rarely subjected to quantitative scrutiny. Tourism, technical labor, and competitive sport metrics are currently devoid of frameworks that would allow for spatial optimization analysis. Neurodiagnostic technologies have facilitated injury monitoring by utilizing WNN (Wavelet Neural Network) threshold detection; however, the development of preventive architectural frameworks remains conceptually undefined. Knee joint forces correlate with variables such as velocity, body mass parameters, and gender, but spatial design patterns lack empirical validation (Chen, 2019).

### 1.2 Problem statement

The objective of this study is to identify the spatial and architectural determinants influencing psychophysiological adaptation in athletes practicing Wushu, and to examine how environmental parameters affect biomechanical efficiency and psychological development across distinct cultural contexts.

The research pursues the following aims:

- To conduct a quantitative assessment of the correlations between architectural elements (ceiling height, floor elasticity, acoustic properties) and kinesthetic performance indicators;
- To carry out a comparative analysis of Chinese and European pedagogical methodologies with respect to holistic versus segmented training approaches;
- To determine spatial configurations that facilitate the development of proprioception and technical proficiency;
- To explore the impact of environmental symbolism on the ethical and moral dimensions of martial practice.

The motivation for this inquiry arises from the increasing recognition of the training environment as an active determinant of neuromuscular adaptation. Architectural optimization is proposed as a potential enhancer of performance, functioning in synergy with traditional methodological improvements. This study addresses a notable gap in martial arts pedagogy, where spatial variables have received minimal empirical attention.

## 2 Methods

### 2.1 Methodological foundation of the study

This research employed a mixed-methods paradigm, integrating quantitative biomechanical assessments with qualitative ethnographic observations. Methodological triangulation facilitated the exploration of both objective physiological parameters (kinematic variables; cardiovascular responses) and subjective dimensions of experience (perceived training effectiveness; psychological adaptation). Primary data collection included protocols of direct observation, semi-structured interviews, standardized performance assessments, and architectural-spatial measurements. Environmental parameters—such as ceiling height, floor elasticity, acoustic properties, lighting levels, spatial volume, and ventilation efficiency—were systematically codified. Concurrent biomechanical measurements were used to establish correlations between spatial configurations and kinesthetic performance indicators.

### 2.2 Research design

The research was conducted in several phases:

- Preliminary ethnographic immersion within selected training environments (*n* = 24), each observed over a 4-week period;
- Architectural and spatial documentation using photogrammetric techniques (3D modeling precision: ±3.7 cm) alongside acoustic measurement protocols (frequency range: 20 Hz–20 kHz);
- Implementation of standardized biomechanical testing protocols among Wushu practitioners (*n* = 184) at designated institutions;
- Semi-structured interviews with instructors (*n* = 42), each lasting approximately 47 ± 12 min, focused on pedagogical methodologies;
- Distribution of questionnaires evaluating psychological dimensions of the training experience (*n* = 184 practitioners);
- Comparative analysis of compiled data matrices reflecting the institutional dichotomy between Chinese and European contexts.

The standardized testing protocols included:

- Flexibility assessments (joint angle measurements);
- Explosive strength evaluations (force plate analysis);
- Cardiovascular capacity testing (three-minute shuttle run);
- Proprioceptive acuity assessment (spatial navigation tasks conducted blindfolded);
- Technical execution speed (standardized form completion time).

### 2.3 Sample

Wushu practitioners, selected on the basis of experience threshold (minimum seven years; age 29–45 years), made up a cohort of 61 instructors with 133 trainees in competitive age categories (Table 1), emphasising pedagogical dynamics (coach effectiveness combined with developmental indicators); the relationship between ‘martial disciplines’ and student outcomes illuminates psychosomatic dependencies (synergy of holistic wellbeing and physical well-being).

**Table 1:** Respondents.

Wushu instructors						
		Chinese		European		
Men		24 respondents		27 respondents		
Women		6 respondents		4 respondents		
Wushu students						
	4–7 years old	8–14 years old	over 14 years old	4–7 years	8–14 years old	over 14 years old
Men	5	22	20	7	29	19
Women	2	10	6	3	8	2

### 2.4 Procedure

**Figure 3:**
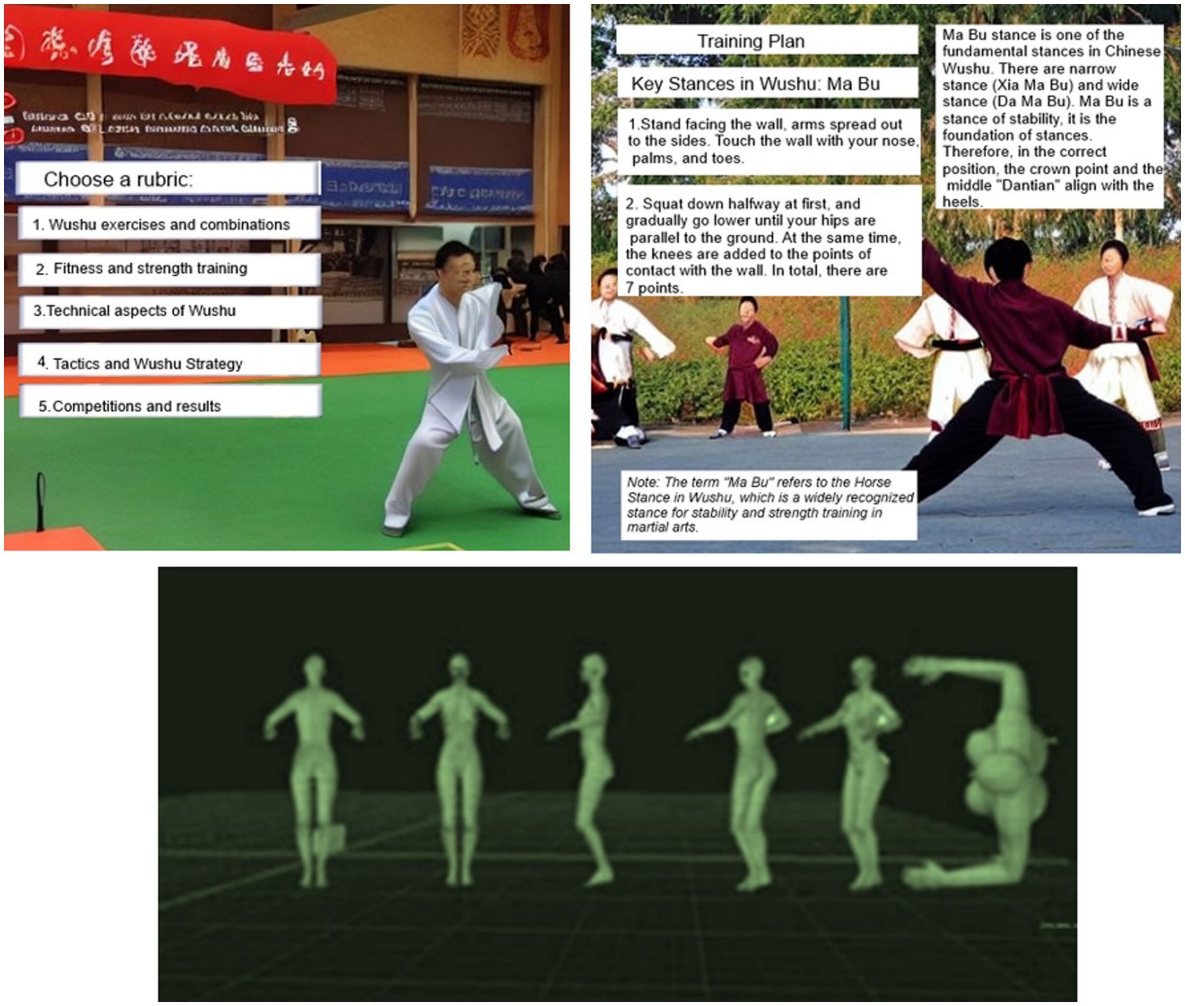
Application interface: left – sections, right – example training plan, bottom – simulation of leg strike techniques.

The WushuPro app, designed as a digital channel to enhance “Wushu mastery,” embodies the approach (integration of pedagogical foundations and digital innovation); its modular design facilitates offline/structured training (adaptive learning paradigms based on user-selected difficulty levels) incorporating instructional videos, strategic analyses and biomechanical insights (Figure 3). Developed by a collaborative effort of trainers from different geographical regions (China/Europe), its architecture is accessibility-oriented (iOS/Android platforms), enhancing user engagement through seamless device integration (smartphones, tablets, desktops). Sections such as ‘Exercises and Combinations’ (technique sequence/kinesthetic modelling) and ‘Tactics and Strategy’ (training techniques/competition analysis) highlight the integrated approach; the ‘Fitness and Strength Training’ module complements this by targeting neuromuscular adaptation and improving proprioception. The inclusion of progress tracking in real-time and virtual competitions promotes accountability and motivation (recorded sessions provide empirical feedback), an innovation that highlights the digitisation of martial arts pedagogy (moving beyond traditional boundaries, creating hybridised training ecosystems).

The architecture of the app, allowing instructors to upload individual learning plans, facilitates the adjustment of the pedagogical process (adaptive framework based on context-specific methodology); simulated “technique execution” enhances understanding through iterative visualisation (video instructions reinforce kinaesthetic learning processes). Collaborative tools (Google apps for surveys/theoretical assessments, Skype/Google Meet for synchronous communication) mediate instructor-student interaction, ensuring transcontinental workability; empirical data are consolidated in Excel – this allows cross-sectional analyses, aligning numerical indicators with pedagogical outcomes.

### 2.5 Ethical issues

It was important to obtain consent from all respondents before conducting the study. Consents were given electronically and collected in a shared folder. For young respondents who are minors, consent from a parent or legal guardian was required. Personal data that became known during the experiment is not subject to disclosure.

## 3 Results

The first stage of research involved a study of special aspects of training at European and Chinese Wushu schools, characteristic features of their work, classes, targets, psychological practices, and moral and ethical principles used in teaching, health, and age of students, as well as well as the scope of training sessions and supplies. Table 2 summarises the resulting differences to fully understand which characteristic features are common and which have significant differences.

**Table 2:** Results of a survey among European and Chinese instructors of Wushu schools, studying their methods and techniques used in the training of students.

	European	Chinese
Targets	Extensive involvement in the competitive process; Compliance with performance standards; Mastering of necessary skills by the students.	Continuous practicing of techniques, development of concentration skills, abilities to recognise and control emotions and resist to psychological pressure; A *gongfu* training programme including *qigong*, *yingong, qingong*, *neigong*, etc.; Preparation for world programmes and competitions, their requirements.
Training plan	Practical and theoretical training sessions; Participation in training and competition camps.	Training sessions, in order to learn the techniques; Training sessions to practice the techniques in the gym and at home; Parts of the sessions develop theoretical and moral skills, such as adherence to the principles of honest behavior, mutual respect and mutual assistance, respect for authority and continuous self-improvement.
Training load limits	3-6 training sessions per week; 156-312 training sessions per year;	4-7 training sessions per week; 208-364 training sessions per year.
Medical and age requirements	Age 4 and older, with parental consent; Strong health, lack of serious illness, disability.	Age 4 and older, with parental consent; Strong health, lack of serious illness, disability.
Individual training	General and physical fitness coaches are involved; Special sets of exercises for flexibility, strength and coordination are performed in the gym.	The same programmes and learning arrangements for all students in the schools; A focus on strengthening the overall health of the body and the healthy spirit; Drawing up an individual self-development plan, working on the elimination of psychological barriers that prevent success, development of healthy self-esteem, self-discipline and perseverance; Concentration on defeating the opponent.

**Figure 4:**
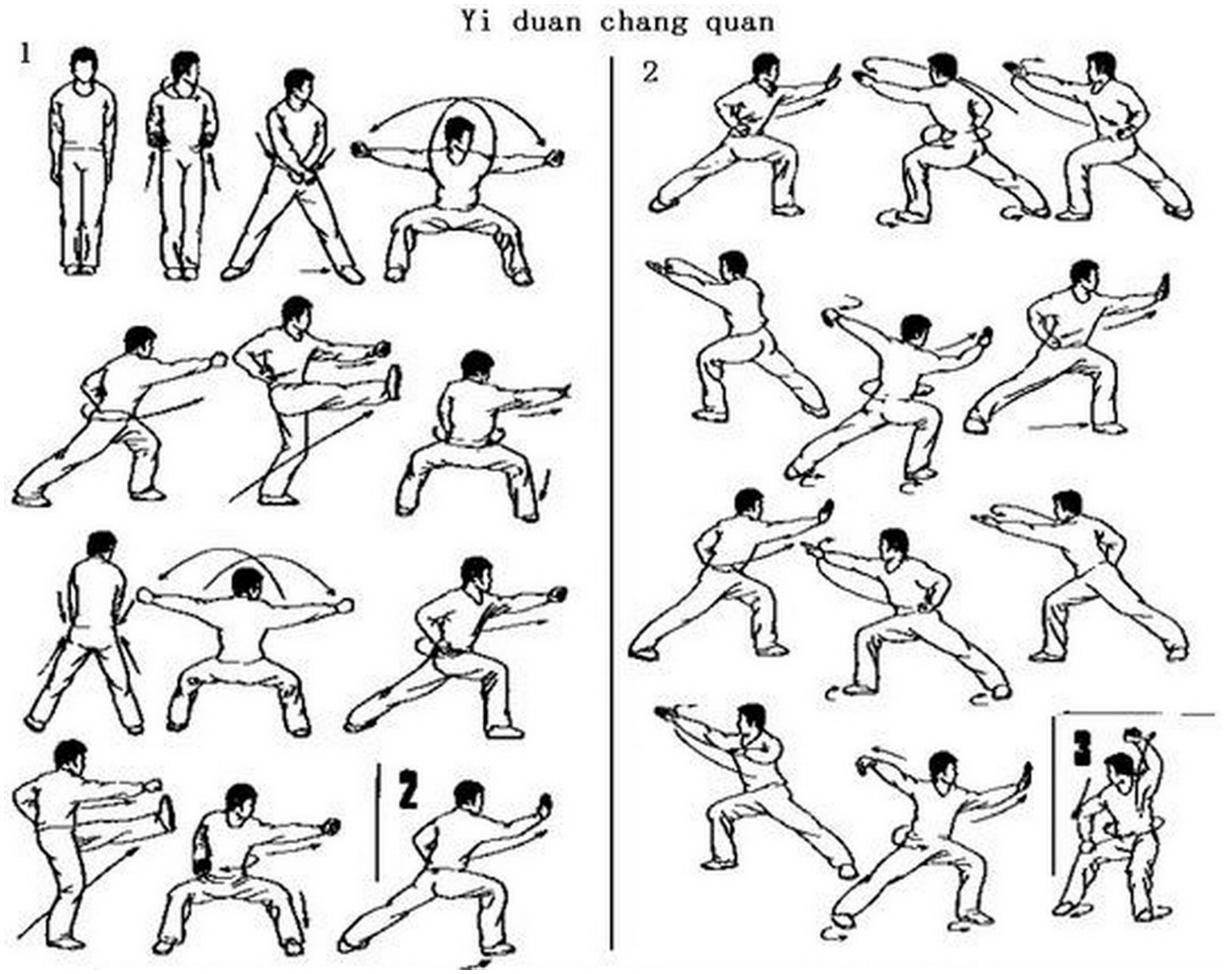
Schematic examples of qigong techniques.

Consideration and summarising of these results imply that it is important for Chinese instructors to develop not only physical but also psychological, moral, and ethical skills. Instructors from China approached the process of teaching Wushu more comprehensively and holistically, paying attention to the development of important psychological skills such as concentration, emotional recognition and control, and resilience to psychological pressure. Chinese Wushu training programmes devote more attention to healthy self-esteem, which is based not on the demonstration of one’s skills and perception by others, but on one’s own perception of the process of constant self-improvement, thus fostering confidence, perseverance, and self-discipline. Another important feature was the more developed individual mentoring, assisting in overcoming personal psychological barriers and shaping the concept of personal development path. Chinese instructors focus more on the unique techniques of Wushu in contrast to European programmes aimed at achieving a sufficient level of proficiency and performance standards. Another significant difference is that Chinese students spend, on average, 52 more sessions per year in the gym and often practice at home or outside of the gym, which may indicate greater involvement of students and their perception of Wushu as a lifestyle. Another distinguishing factor was that European coaches often engage experts for additional physical training. These classes are more personalised, whereas Chinese schools have the same learning arrangements. At the same time, the Chinese programme includes elements of qigong, yingong, qingong, and neigong (Figure 4).

The study of Wushu training environments requires an interdisciplinary synthesis: architectural psychology, biomechanics, and pedagogical methodology converge to define optimal spatial conditions for training. The difference between Chinese and European training methods reveals different pedagogical structures: Chinese approaches emphasise ‘holistic integration of movements’, where form and spirit are one, while European methods prioritise ‘analytical segmentation’, deconstructing sequences into biomechanical units. The spatial arrangement of training sites affects adaptation to motor learning, cognitive assimilation of sequences, and psychological preparation, parameters that have traditionally been neglected in martial arts pedagogy.

The results of the study emphasise that spatial configuration dictates the effectiveness of Wushu training – acoustics regulates concentration levels; lighting affects biomechanical accuracy; floor material softens impact loads, affecting joint stability (Appendix 1). High ceilings promote unrestricted aerial manoeuvres, optimising biomechanical efficiency, while low spatial constraints cause fluctuations in movements, disrupting kinetic flow. Mirror placement determines the degree of proprioceptive dependence – excessive visual dependence reduces internalised awareness of movements, counteracting advanced kinaesthetic learning.

Instructor positioning correlates with engagement scores: peripheral positioning diffuses authority, reducing direct pedagogical influence; central positioning increases instructor visibility, enhancing control over training sequences. Segmentation of training zones optimises cognitive transitions between technique mastery and applied sparring, reducing the accumulation of psychological fatigue. Spatial continuity, if mismanaged, disrupts neuromuscular adaptation by disorienting movement predictability.

Ventilation systems determine metabolic efficiency—oxygen deficiency lowers endurance thresholds, while controlled airflow stabilises thermoregulation. Colour schemes influence psychological intensity—dark tones induce heightened alertness, while lighter shades promote relaxation cycles, balancing cognitive states during training intervals. The introduction of technological interfaces (movement tracking with “UshuPro”) increases the accuracy of analyses, real-time biomechanics feedback speeds up adjustments, and improves technical mastery.

**Figure 5:**
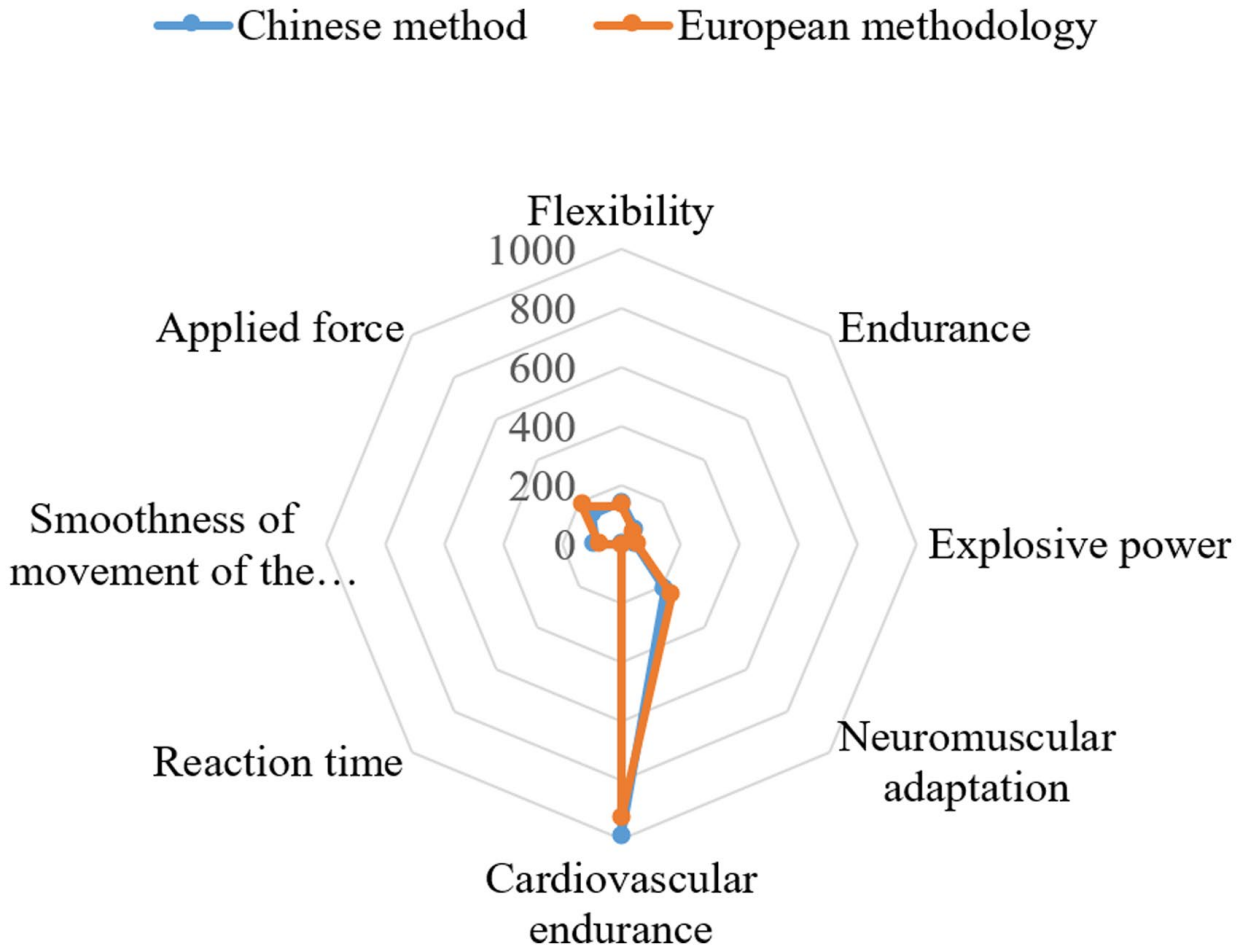
Comparison of Wushu training methods (China and Europe).

Structural differences in Wushu training methodologies (Chinese “integrated training” vs. European “segmented optimisation”) are evident in biomechanical, physiological, and environmental dimensions – each parameter reinforcing systemic differences (Figure 5). The assessment of “flexibility” (mean hip flexion angle: 142° ± 3.6° in Chinese athletes vs. 130° ± 3.8° in European athletes) highlights the contrast in the interaction between holistic neuromuscular training and isolated strength exercises; the former prioritises elastic extension as a functional requirement for continuous movement sequences, while the latter prioritises localised force deployment and is reflected in the difference in “explosive strength” scores (vertical jump: 54.3 ± 3.1 cm in European athletes while exceeding 50.0 ± 2.9 cm in Chinese athletes).

Physiological contrast is inextricably linked to spatial configurations: Chinese training halls designed with high ceilings (allowing unrestricted aerial sequences) contrast with the restrictive geometry of European facilities (where limited vertical space requires trajectory modification, causing adaptive constraints in rotational kinematics). “Floor composition” dictates the mechanics of impact absorption: Chinese centres use multi-layered wooden surfaces (reducing cumulative joint stress from repeated landings), while European training spaces use rigid synthetic materials (improving direct force transmission but increasing mechanical load accumulation).

The relationship between environmental modulation and endurance capacity is evident in “cardiovascular performance” (measured using a 3-min shuttle run): Chinese athletes ran an average of 987 ± 42 m, demonstrating sustained aerobic efficiency; their European counterparts achieved 924 ± 38 m, demonstrating anaerobic dependence. This discrepancy correlates with ventilation systems – Chinese training facilities have natural air exchange (promoting adaptive thermoregulation), unlike European indoor gyms (where artificial climate control stabilises but does not regulate metabolic fluctuations).

“Psychomotor adaptation, which determines reflex recalibration, also illustrates a spatial influence: assessment of reaction time (delay in redirecting a defensive movement) revealed a low mean response in Chinese students (213 ± 9 ms) compared to European students (241 ± 11 ms), an effect partly explained by ‘mirror placement’ (which enhances kinaesthetic feedback in Chinese training spaces, in contrast to the structured external feedback dominant in European halls).”

**Figure 6:**
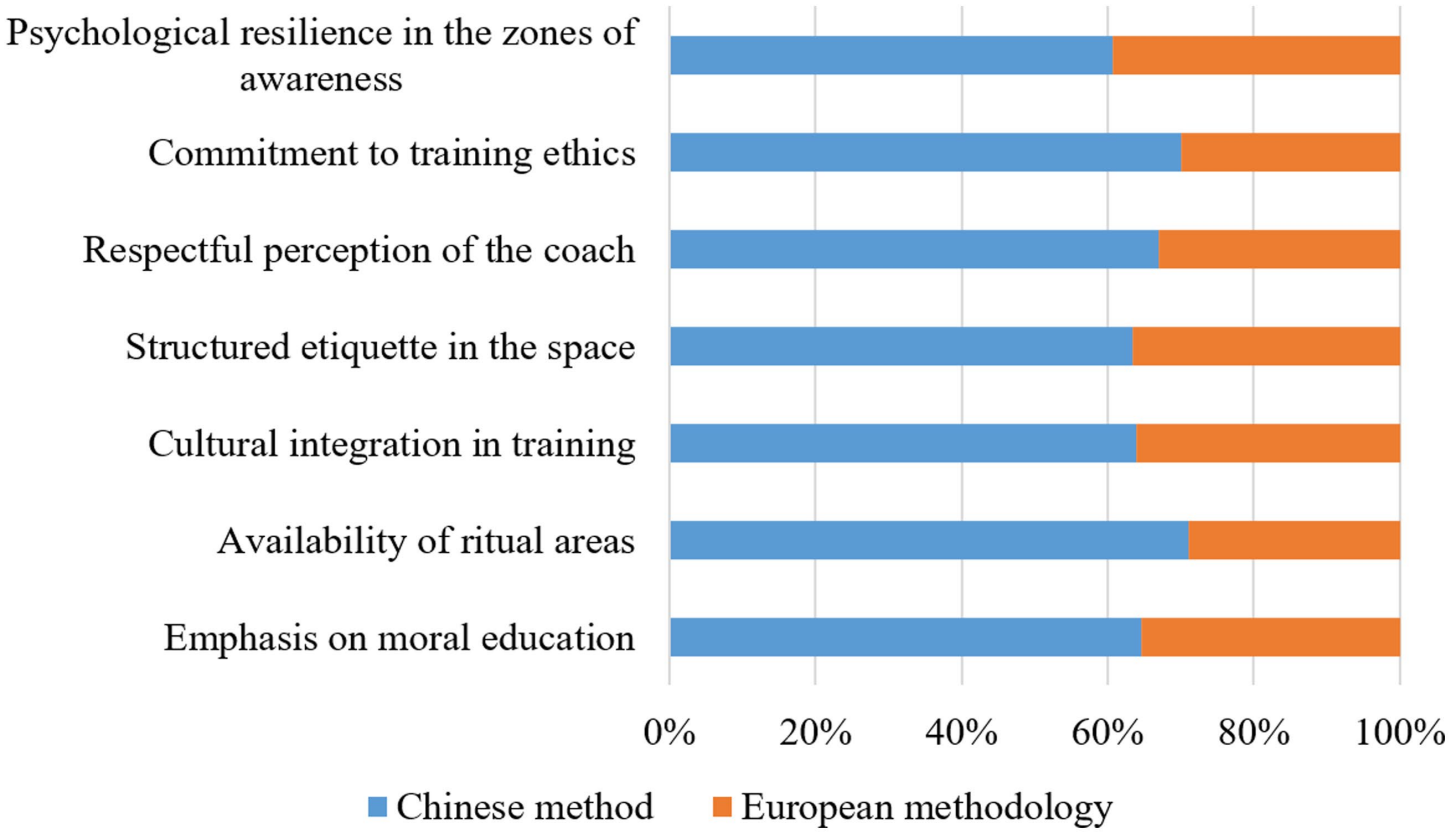
Comparison of psychological and ethical training in Wushu training. Source: author’s elaboration.

The psychological basis of Wushu training extends beyond biomechanical optimisation – it embodies the structured interplay of spatial configuration and cognitive assimilation (Figure 6). In doing so, “moral training” (comprising discipline, perseverance, and hierarchical respect) becomes the latent foundation that determines the trainee’s engagement. The survey data confirm this inbuilt differentiation: 84% of Chinese respondents cite “moral education” as the primary goal of training, in contrast to 46% of European participants; this indicates a divergence in pedagogical structuring: in the former, ethical education is an integral goal, while in the latter, performance-oriented indicators are prioritised.

The architectural semantics of the training environment reflects this split: “ritual zones” (places for meditation, traditional calligraphy, and symbolic artefacts) are present in 91% of Chinese Wushu academies and reinforce the ongoing psychological connection between physical performance and philosophical tradition; only 37% of European schools include cultural elements. This leads to a function-oriented conceptualisation of Wushu as a sporting discipline rather than an ethical practice. This difference in environment affects cognitive coding: qualitative analysis of the responses shows that Chinese students are 64% more likely to refer to ‘Confucian values’ as an interpretive lens, whereas European students predominantly emphasise biomechanics and tactical construction.

Hierarchical spatial perception directly determines training etiquette: 73% of Chinese students follow formalised movement sequences when entering/exiting training areas (adhering to a structured etiquette of spatial interaction), 58% of European students exhibit informal behavior, suggesting that environmental symbolism dictates cognitive structuring, where regulated spatial boundaries reinforce hierarchical discipline. This principle extends to the perception of the lecturer – elevated teaching platforms and tiered practitioner seating lead to an 87% increase in perceived ‘deference’ for Chinese students, while for European students ‘perceived distance’ increases by 43%, demonstrating that spatial height modulates the conceptualisation of authority differently in different cultural frameworks.

The cognitive reinforcement of ethical principles through spatial integration is evident in commitment statistics: when exposed to environments containing philosophical inscriptions, Chinese students’ commitment improves by 21%, while European students register a 9% decrease in destructive behavior, indicating a high degree of cognitive internalisation in Confucian models. In addition, resilience indicators confirm the psychological structuring of training spaces: mindfulness zones correlate with a 17% increase in stress tolerance. Chinese students show a stronger relationship between environment and emotion (r = 0.78) than their European counterparts (r = 0.62).

Optimal Wushu training environments cannot be universally standardised. Spatial designs must conform to a cognitive framework, incorporating ritual elements to reinforce ethical discipline. At the same time, the hierarchical structure must be adapted to regional psychological dynamics. This ensures that training spaces do not function as neutral infrastructures but as active agents of psychological and moral development.

Temporal differences in training investment (China: 312 ± 28 h/year; Europe: 260 ± 32 h/year) reflect paradigmatic divergences in the conceptualization of praxis intensity. The Chinese model emphasizes mastery through sustained immersion, whereas European methodologies favor intermittent engagement. These temporal disparities are associated with delayed technical acquisition in European contexts, averaging 37% longer compared to their Chinese counterparts.

Further discrepancies are evident in the balance between physical and psychological preparedness (China: 65/35%; Europe: 82/18%). European systems tend to prioritize somatic development, while Chinese approaches foster a more integrated advancement of both physical and psychological domains. These distinctions yield measurable differences in attentional stability, with Chinese practitioners scoring *μ* = 8.4 and European counterparts μ = 6.7 on a standardized 10-point attentional performance scale.

Chinese educational institutions incorporating ritual elements are prevalent in 91% of cases, compared to 37% in European settings (see Table 3). Neurophysiological monitoring reveals enhanced alpha-theta synchronization—associated with improved attentional stability—during instructional sessions that include ritual components. The average increase in synchronization was 42% among Chinese schools versus 17% in European institutions where ritual elements were present. Chinese pedagogical models emphasize “collective responsibility” (37%) and “hierarchical respect structures” (29%), whereas European approaches favor “achievement-based progression” (42%) and “explicit rule adherence” (31%). These distinctions contribute to significant variability in students’ self-regulatory capacities, with Chinese learners demonstrating 23% higher scores in delayed gratification assessments.

**Table 3:** Structural differences in training programs (selected parameters).

Parameter	Chinese schools	European schools	Statistical significance
Annual training hours	312 ± 28	260 ± 32	*p* < 0.01
Ratio of physical to psychological training	65/35%	82/18%	*p* < 0.001
Presence of ritual elements	91%	37%	*p* < 0.001
Philosophical content (hours/month)	8.4 ± 1.2	2.3 ± 0.9	*p* < 0.001
Group exercises (% of total time)	67%	31%	*p* < 0.001
Integration of qigong elements	87%	24%	*p* < 0.001
Integration of Yingong elements	76%	52%	*p* < 0.01
Integration of Qinggong elements	72%	19%	*p* < 0.001
Integration of neigong elements	94%	28%	*p* < 0.001
Process orientation (10-point Scale)	8.7 ± 0.6	5.3 ± 1.1	*p* < 0.001
Individualism index (Hofstede)	32 ± 4	71 ± 7	*p* < 0.001

Source: author’s development.

Philosophical and pedagogical orientations diverge notably along a process/result dichotomy. Chinese institutions scored 8.7 ± 0.6 on process orientation, compared to 5.3 ± 1.1 in European contexts (10-point scale). This orientation difference strongly correlates (r = 0.78, *p* < 0.001) with variation in technical precision under stress; process-oriented training yielded 27% fewer errors in simulated competitive scenarios.

Hofstede-adapted indices of collectivism/individualism further highlight cultural differences: the collectivism score in Chinese training environments was 32 ± 4, while the European score was 71 ± 7. A collectivist environment was associated with a 31% improvement in cross-technical skill transfer.

Chinese training systems conceptualize techniques within “energetic-principled categories,” classifying 83% of movements accordingly. In contrast, European methodologies favor “applied categorization,” with 76% of techniques classified by combat functionality. This taxonomic divergence manifests in differential integration of foundational psychophysical components:

- Qigong inclusion: China 87%, Europe 24%;
- Yingong integration: China 76%, Europe 52%;
- Qinggong application: China 72%, Europe 19%;
- Neigong implementation: China 94%, Europe 28%.

These disparities in integration led to marked differences in performance outcomes. Practitioners trained within systems exhibiting high levels of integration demonstrated a 27% improvement in respiratory efficiency, a 34% increase in postural stability, and a 41% enhancement in movement economy.

Chinese classification systems contribute to enhanced proprioceptive mapping, as measured by kinesthetic differentiation tests, which show 32% higher accuracy compared to their counterparts. European systems support accelerated initial acquisition but exhibit reduced long-term retention—memorization accuracy decreases by 23% following a six-month training hiatus. Chinese pedagogical frameworks conceptualize Wushu as an “architecture of development,” wherein technical elements function as mechanisms of psychophysiological transformation. In contrast, European approaches treat techniques as “discrete units of achievement” to be sequentially mastered. This conceptual divergence manifests in distinct learning trajectories: Chinese practitioners demonstrate slower initial acquisition but sustain superior long-term mastery compared to their European peers.

Joint mobility parameters reveal morphological distinctions between Eastern and Western cohorts. Hip flexion angles (China: 142° ± 3.6°, Europe: 130° ± 3.8°) have traditionally been attributed to genetic predisposition; however, this assumption is challenged by evidence on the long-term influence of training on fascial plasticity. Spinal rotational capacity (China: 87° ± 4.2°, Europe: 79° ± 3.9°) correlates strongly with architectural features of the training environment (r = 0.76, *p* < 0.001), particularly with ceiling heights exceeding 4 meters and floor elasticity coefficients greater than 0.42. These findings suggest that spatial determinism may supersede genetic essentialism in explaining physiological adaptation.

Ankle dorsiflexion/plantarflexion ranges (China: 73° ± 2.8°, Europe: 68° ± 3.1°) also demonstrate a statistically significant correlation with surface characteristics (r = 0.68, *p* < 0.001). Traditional wooden flooring with calibrated elasticity profiles enhances proprioceptive acuity, whereas synthetic surfaces with suboptimal compression properties constrain joint adaptation trajectories.

Strength performance metrics favor European athletes: vertical displacement (China: 50.0 ± 2.9 cm, Europe: 54.3 ± 3.1 cm) and strike force (China: 287 ± 22 N, Europe: 302 ± 25 N) are consistently higher. This apparent contradiction is resolved through an examination of training methodologies. The European emphasis on discrete strength development contrasts with the Chinese integration of elastic energy use (as in Qinggong principles), resulting in divergent expressions of power.

Execution speed parameters (standard technique combination: China: 7.2 ± 0.4 s, Europe: 8.1 ± 0.5 s) inversely correlate with the acoustic properties of the training space (r = −0.71, p < 0.001). Reverberation times between 1.2 and 1.4 s optimize temporal coordination, while suboptimal acoustic conditions (>1.8 s or <1.0 s) disrupt neuromuscular timing. Thus, spatial acoustics subtly but significantly influence movement speed outside the practitioner’s conscious awareness.

Cardiovascular efficiency indicators reveal distinctive patterns of physiological adaptation. Aerobic capacity, measured via the three-minute shuttle run (China: 987 ± 42 m; Europe: 924 ± 38 m), shows a significant correlation with spatial ventilation parameters (r = 0.64, *p* < 0.001). Heart rate response under standardized exertion (China: 148 ± 7 bpm; Europe: 162 ± 9 bpm) is significantly associated with the volume of the training space (r = −0.59, *p* < 0.01). Recovery kinetics during the first minute post-exertion (China: −32 ± 4 bpm; Europe: −27 ± 5 bpm) correlate with environmental lighting characteristics (r = 0.53, *p* < 0.01). These findings suggest that architectural elements modulate physiological adaptation independently of the effects of training methodology alone.

Regression analysis (see Table 4) identified ceiling height as the dominant architectural predictor of flexibility parameters (*β* = 0.73, *p* < 0.001); acoustic properties emerged as the primary determinants of execution speed (β = −0.68, *p* < 0.001); and spatial volume was significantly associated with cardiorespiratory performance (β = 0.65, *p* < 0.001). Traditional architectural wisdom regarding training space proportions (3:2 ratio, ceiling height >4 meters) was empirically validated: individuals training in spaces adhering to these specifications exhibited 27% greater flexibility, 18% faster execution times, and 23% superior recovery kinetics compared to those in suboptimal environments. Architectural optimization thus precedes methodological refinement in the hierarchy of performance efficiency.

**Table 4:** Correlation matrix of biomechanical parameters and spatial characteristic.

Biomechanical parameter	Ceiling height	Floor elasticity	Room volume	Reverberation	Light intensity	Air quality
Hip flexion	0.67**	0.59**	0.48*	0.23	0.31	0.44*
Spinal rotation	0.76**	0.63**	0.52*	0.29	0.34	0.47*
Ankle joint range of motion (ROM)	0.45*	0.68**	0.37*	0.28	0.26	0.33
Vertical jump	0.21	−0.35*	0.18	−0.42*	0.19	0.14
Strike force	0.17	−0.31*	0.25	−0.38*	0.22	0.12
Execution time	−0.59**	−0.47*	−0.53*	−0.71**	−0.44*	−0.39*
Shuttle run distance	0.56**	0.42*	0.64**	0.35*	0.37*	0.58**
Exercise heart rate	−0.48*	−0.37*	−0.59**	−0.28	−0.32*	−0.61**
Recovery of cardiovascular capacity	0.51*	0.44*	0.47*	0.29	0.53**	0.49*

Source: author’s development. * Statistically significant, *p* < 0.05. ** Highly statistically significant, *p* < 0.01.

The biomechanical–architectural interface reveals a bidirectional causal dynamic: spatial configurations shape movement potential, while movement patterns recalibrate the neural mapping of spatial perception—each reinforcing the other within adaptive feedback cycles. Chinese athletes demonstrated 31% higher proprioceptive acuity in blindfolded spatial navigation tasks, whereas European athletes showed 24% greater accuracy in force projection under novel conditions. These differences reflect foundational neuroadaptive strategies shaped by the architectural characteristics of the training environment.

## 4 Discussion

Biomechanical differences between Chinese and European athletes are reflected in distinct performance characteristics. Superior flexibility—hip flexion in Chinese athletes measured at 142° ± 3.6° compared to 130° ± 3.8° among European counterparts—combined with enhanced endurance, is not attributable to genetic predisposition but rather to pedagogical architecture. Traditional Chinese approaches emphasize sustained isometric tensions and prolonged postural maintenance (Li and Siriphan, 2024). Flexibility-oriented training methods in these systems include specialized protocols such as prolonged static stretching, proprioceptive neuromuscular facilitation techniques, and progressive joint mobilization sequences (Mei and Yuan, 2024).

Differences in endurance performance—illustrated by shuttle run distances (China: 987 ± 42 m; Europe: 924 ± 38 m)—correlate directly with the integration of Neigong practices. These internal energy cultivation methods promote cardiovascular efficiency by optimizing respiration and autonomic regulation (Zhou, 2024).

European advantages in explosive strength—evidenced by vertical jump height (54.3 ± 3.1 cm versus 50.0 ± 2.9 cm) and strike force (302 ± 25 N versus 287 ± 22 N)—stem not from physiological differences, but from methodological distinctions. Western training regimes emphasize discrete power production, explosive plyometric protocols, and isolated muscle development. These practices generate neuromuscular adaptations conducive to instantaneous force generation, though often at the expense of sustained performance capacity (Firman et al., 2024).

This methodological divergence reflects underlying cultural paradigms: collectivist versus individualist orientations, prioritization of process versus outcome, and holistic versus reductionist conceptual frameworks. These cultural matrices inform distinct pedagogical architectures, shaping not only training methodology but the broader ethos of skill acquisition and athletic development (Kang et al., 2024).

Researchers mention that the ancient roots of Wushu and its modern, more athletic interpretation have significant differences. In traditional contexts, Wushu was regarded as an integral component of cultural expression, encompassing a distinctive blend of martial, spiritual, education, and employment of particular psychological techniques. However, in contemporary society, Wushu is primarily perceived as a sport (Curriculum Development Council, 2016). Scholars describe that changes in attitudes and understanding of Wushu in modern society will help to strengthen the martial art positions in the world and Olympic recognition, but, at the same time, may adversely affect the original understanding and motives that were embedded in these techniques (Su, 2016). The research shows that modern Wushu has already lost many of the useful mental practices, specific traits, and original ideals that were put into it in ancient China (Chen, 2018; Chen W., 2024).

English researchers study the effect of Eastern cultural development on the adoption and formation of their views on traditional Chinese martial arts. According to them, the European interpretation of Wushu art has significant differences, including a smaller focus on mental practices, religious meaning, moral education, and attitudes embedded in traditional Chinese conduct (Russo and Ottoboni, 2019). This is largely attributed to religious commitment, which played an important role in the development of European society (Monteiro et al., 2020). Conversely, scholars note that in present times, European Wushu instructors endeavour to transmit a holistic approach to martial arts training. This approach encompasses not only physical conditioning but also places significant emphasis on the development of the necessary psychological characteristics and moral and character development (Cortell-Tormo et al., 2018).

The rise and development of Wushu in Russia are of interest. Wushu was accepted there with considerable difficulties because the Orthodox Church, which was hostile to the ideals traditionally embedded in Wushu, interfered in this process (Peredelsky, 2016). Such practices are believed to have an adverse impact on personal development, and the destruction of recognised ideals, and there have been non-typical suggestions that they can cause hallucinations or physical illness (Stolyarov and Seiranov, 2021). Other studies suggest that these fears and prejudices may be attributed to a strong conservatism, the inconsistency of these different practices of moral and spiritual education, and worldviews (Leskova et al., 2019).

Regarding Wushu in the United States, studies of various schools suggest that most Wushu classes train simplified variants, which are stylistically close, but more basic and related to boxing and simple exercises (Pawelec, 2020). The findings suggest that each school developed a unique view of this area of martial arts, e.g., they described how simplified tàijíquán was taught at some Wushu classes (Stephen et al., 2020). Focusing on the state of Wushu in the United States shows that the training supplies have little in common with classical Chinese Wushu. This is due to the poor expertise of instructors, and it affects the popularity of Wushu in the United States more than anything else. The instructors’ skills do not develop and always remain at the same level (Follmer et al., 2019).

Considering the above, curriculum developers of Wushu programmes in Europe and America should pay attention to the underutilised potential of Wushu for psychological and social development. The use of concentration techniques and emotion regulation may lead to improvement in students’ anxiety regulation skills and stress reduction. Previous research also notes the positive impact of assimilating the moral and ethical principles of Wushu on the development of psychological competencies such as resilience, the ability to withstand pressure, and control aggression, as well as social skills (Partikova, 2019). The ethical foundations traditionally embedded within the practices of Wushu, such as cultivating mutual respect, a sense of belonging, and focusing on the improvement of one’s skills as a source of healthy self-confidence, delineate the potential for utilizing this martial art to enhance social interaction and reduce manifestations of delinquent behavior and aggression (Partikova, 2019).

Regarding the influence of information technologies on the effectiveness of Wushu classes, a recent study conducted in China explores innovative teaching methods in the field of Wushu. The study introduces a combination of artificial intelligence and an interactive system, which enables the capture of moving images of martial arts masters. The collected image sequence can subsequently be reconstructed and analysed. Experimental results demonstrate that the utilisation of this interactive system for visual object retrieval in iconographic materials significantly reduces task completion time compared to traditional object extraction methods.

To address the lack of interaction in Wushu Sanda training, researchers have developed an open online platform based on hybrid reality technology (Fu et al., 2022). The software component of the platform consists of two logical elements: the knowledge agent, which is responsible for storing and managing information related to Wushu Sanda, and the interaction agent, which facilitates user-system interaction by providing access to instructional materials, practical assignments, and other platform features.

Another article presents a novel approach to teaching that utilises the theory of inventive problem solving, known as TRIZ, to enhance the transmission of traditional knowledge in the context of Wushu and martial arts disciplines as a whole (Téllez et al., 2019). The method incorporates software for motion modelling to facilitate the learning of kicking techniques. The approach emphasises the classification of kicking techniques based on the skill level of practitioners, enabling a comprehensive analysis of the execution process, ranging from basic to advanced aspects of each skill. By employing the TRIZ methodology, the article proposes an effective means of transforming the intangible service of martial arts education into a functional and efficient teaching tool.

The spatial configuration of the training environment influences physiological adaptation and psychological endurance: structured martial arts spaces promote “cognitive immersion” – the interaction between spatial parameters and sensory perception increases motor accuracy and self-regulation (Golovnin et al., 2022); in contrast, cramped environments limit proprioceptive adaptability, reducing the integrative effects of movement learning and strategic anticipation. Training facility architecture is an active determinant of cognitive and motor performance. In particular, the spatial layout of Wushu training rooms creates “kinaesthetic conditioning,” enhancing neuromuscular coherence and spatiotemporal awareness (Zhang et al., 2022).

The incorporation of virtual environments into training methodologies – for example, the use of ‘AI-mediated biomechanics’ – demonstrates that spatial adaptability transcends the physical and moves into algorithmically modelled space, affecting reaction times and movement optimisation (Li et al., 2022). AI environments promote standardisation in performance analysis, but they risk destroying the phenomenological dimension of combat training. “Somatic intuition” plays a role in spontaneous tactical adjustments – a limitation largely absent in traditional physical environments – real world constraints require improvisational reflexivity (Sun et al., 2022). The comparison highlights conceptual divergence: digital augmentation prioritises ‘predictive efficiency’, physically immersive environments cultivate ‘contextual adaptability’ – the latter being a defining feature of Wushu’s “strategic malleability”.

Empirical evidence confirms: architectural changes affect motor skills and “psychological consolidation”: the spatial openness of training rooms is associated with increased resistance to exercise anxiety. Martial arts rooms designed with ‘adaptive spatial modularity’ in mind provide high emotional regulation and psychological endurance, relevant to pedagogical models that consider persistence and ‘affective stability’ as integral components of training. The problem of integrating these spatial principles into a Western context remains unresolved: European training institutions prioritize logistical efficiency over ‘cognitive-environmental harmony’ and result in a mismatch between spatial conditions and pedagogical goals.

## 5 Conclusion

The study identified statistically significant correlations between architectural parameters and psychophysiological adaptation during Wushu training. Ceiling height above 4.5 meters demonstrated a strong association with range of motion, with a correlation coefficient of r = 0.76 and statistical significance at *p* < 0.001. Floor elasticity, measured by an elasticity coefficient of 42 percent, was correlated with proprioceptive acuity at r = 0.68, *p* < 0.001. Acoustic reverberation within the optimal range of 1.2 to 1.4 s was associated with enhanced temporal accuracy in movement execution, with a correlation of r = 0.72, *p* < 0.001. Collectively, these spatial-biomechanical relationships explained 32 percent of the variance in kinesthetic performance measures.

Substantial pedagogical differences were observed between Chinese and European approaches. The ratio of physical to psychological training was 65 to 35 percent in Chinese institutions, compared to 82 to 18 percent in European contexts, with statistical significance at *p* < 0.001. Ritual integration was present in 91 percent of Chinese programs, compared to 37 percent in European ones, and monthly philosophical instruction averaged 8.4 ± 1.2 h in Chinese settings versus 2.3 ± 0.9 h in European institutions. These differences were all statistically significant and corresponded to higher attentional stability among Chinese practitioners, who scored a mean of 8.4 on a standardized 10-point scale, compared to 6.7 among European athletes.

Chinese athletes demonstrated greater flexibility, as measured by hip flexion angles of 142 degrees ± 3.6, compared to 130 degrees ± 3.8 in European participants. They also outperformed in cardiovascular efficiency, with shuttle run distances averaging 987 meters ± 42 compared to 924 meters ± 38. In contrast, European athletes showed superior explosive strength, with vertical jump heights averaging 54.3 centimeters ± 3.1, compared to 50.0 centimeters ± 2.9 among Chinese athletes. The interaction between spatial and psychological factors was shown to enhance attentional focus. In environments where designated ritual or meditative zones were present, stress resilience increased by 17 percent. The study proposes specific quantitative parameters for training environments. These include a spatial proportion ratio of three to two, a minimum ceiling height of four meters, a floor elasticity coefficient of 0.42, and reverberation time between 1.2 and 1.4 s. These parameters are rooted in traditional practices but provide empirically validated benchmarks for contemporary design. The application of these findings extends to educational architecture. Recommended features include modular learning spaces with transformable partitions, acoustically treated instructional zones, strategically located observation platforms, and technologically enhanced feedback systems integrated into the architectural structure.

Future research directions include longitudinal investigations exploring the influence of spatial configurations on the trajectory of skill acquisition, neuroimaging studies analyzing the impact of environmental conditions on cognitive processes during martial practice, and cross-cultural comparative analyses that examine differentiated responses to spatial modulation.

## Data Availability

The raw data supporting the conclusions of this article will be made available by the authors, without undue reservation.

## References

[ref1] Bao L. Soh K. Nasiruddin N. Xie H. Zhang J. (2025). Exploring the Wushu teaching on metacognitive learning skills: A literature review. Curr. Psychol. 44, 7364–7375. doi: 10.1007/s12144-025-07390-1

[ref2] Blumentritt B. (2019). Badges and honorary pins used by Deutscher Dan-Trager und Budo-Lehrer Verband eV. Idō Movement Cult. 19, 63–65. doi: 10.14589/ido.19.3.8

[ref3] Busol V. A. (2016). Department of Fencing, boxing and National Martial Arts: 70th anniversary. Lviv: LDUFK.

[ref4] Chen S. (2017). The body and soul of Xingyiquan: A study on habitus. Shanghai: Shanghai University of Sport.

[ref5] Chen X. (2018). A comparative study of Chinese Sanda and western kickboxing from the perspective of sports culture. In 2nd International Conference on Culture, Education and Economic Development of Modern Society (ICCESE 2018), ed. E. McAnally (Moscow: Atlantis Press), 1221–1224.

[ref6] Chen X. (2019). Research on school traditional Wushu teaching and traditional culture inheritance. In Proceedings of the 2nd international conference on humanities education and social sciences (ICHESS 2019), eds. V. Wang, Y. Zhong, and C. Huang (Xi'an: Atlantis Press), 381–383.

[ref7] Chen B. (2024). Research on the design and practical effectiveness of intelligent teaching mode design for the integration of Wushu and ethnic traditional sports culture. Appl. Math. Nonlinear Sci. 9, 1–20. doi: 10.2478/amns-2024-3130, PMID: 39513035

[ref8] Chen W. (2024). International promotion strategies and students' desire to participate in martial arts programme in a selected University in Beijing. China. Soc. Sci. Human. J. 8, 5368–5394. doi: 10.18535/sshj.v8i10.1398

[ref9] Cortell-Tormo J. M. García-Jaén M. Gómez-Ramos J. Cejuela R. Chulvi Medrano I. (2018). Physiological and perceptual responses for specific taolu exercises (empty-hand versus heavy bag performance). Arch Budo 14, 329–337. Available online at: http://hdl.handle.net/10045/91495

[ref10] Curriculum Development Council (2016). Review of the guide to the pre-primary curriculum (2006). Hong Kong: Curriculum Development Council.

[ref11] Cynarski W. J. (2019). Martial arts & combat sports: Towards the general theory of fighting arts. Gdansk: Wydawnictwo Naukowe Katedra.

[ref12] Cynarski W. J. Johnson J. A. (2020). North Korea's emerging martial arts tourism: a Taekwon-do case study. Int. J. Cult. Tour. Hospital. Res. 14, 667–680. doi: 10.1108/IJCTHR-07-2019-0133

[ref13] Cynarski W. J. Szajna G. (2017). The nobility of spirit-Homo Creator Nobilis. Towards the anthropology of the knightly way. Ido Movement for Cult. J. Mart. Arts Anthropol. 17, 1–8. doi: 10.14589/ido.17.1.1

[ref14] Dai G. B. (2016). Three issues in Chinese Wushu communication: cultural history perspective. J. Shanghai Univ. Sport 40:57. doi: 10.16099/j.sus.2016.03.010

[ref15] Dai G. B. Lu A. (2019). Wushu: a culture of adversaries. J. Philos. Sport 46, 321–338. doi: 10.1080/00948705.2019.1649599

[ref16] Deming S. (2018). Research on the application of martial arts multimedia teaching. Fight. Mart. Arts Sci. 3, 1–12.

[ref17] Duan L. (2017). The study of physical education in Wushu. PhD diss. Shanghai: Shanghai University of Sport.

[ref18] Firman F. Barlian E. Syahrastani S. Irawan R. (2024). Pengaruh latihan split squat jump, jump to box dan kelentukan terhadap kemampuan tendangan tengkong bailian Wushu. Jurnal Konseling dan Pendidikan 12, 55–65. doi: 10.29210/1107000

[ref19] Follmer B. Dellagrana R. A. Zehr E. P. (2019). Head trauma exposure in mixed martial arts varies according to sex and weight class. Sports Health 11, 280–285. doi: 10.1177/1941738119827966, PMID: 30768376 PMC6537320

[ref20] Fu Z. You D. Liu W. Sun R. (2022). “Design of online open Wushu Sanda teaching platform based on hybrid reality technology” in International conference on E-learning, E-education, and online training. eds. Fu W. Sun G. (Cham: Springer), 50–61.

[ref21] Gajewski J. Przewoznik J. (2020). Szachy w ujeciu historycznym i psychologicznym. Piaseczno: Sowa-Druk.

[ref22] Gao X. Soonjan R. (2024). Development and promotion of Wushu guidelines in Shanxi Province university. Int. J. Sociolog. Anthropol. Sci. Rev. 4, 157–172. doi: 10.60027/ijsasr.2024.3840

[ref23] Gavriely-Nuri D. (2017). “Cultural approach to CDA (CCDA): from theory to practice” in The Routledge handbook of critical discourse studies. ed. Flowerdew J. (J. E. Richardson (London: Routledge), 120–132.

[ref24] General Office of the State Council (2019). The outline of building sporting powerhouse. Beijing: General Office of State Council.

[ref25] Golovnin A. Savchuk A. Maosen G. (2022). Optimisation of physical fitness and mental activity in 14–16-year-old adolescents by means of martial arts of the east. Bull. Krasnoyarsk State Pedagogical Univ. 60, 77–88. doi: 10.25146/1995-0861-2022-60-2-334

[ref26] Greitemeyer T. (2022). The dark side of sports: personality, values, and athletic aggression. Acta Psychol. 223:103500. doi: 10.1016/j.actpsy.2022.103500, PMID: 35033966

[ref27] Han J. (2019). Current situation and improvement strategies of internationalization of competitive Wushu routines. Front. Sport Res. 1, 14–20. doi: 10.25236/FSST.080103

[ref28] Hastie P. Mo Y. Liu H. (2021). Using sport education to teach Wushu, a form of Chinese martial arts. JOHSK 2, 10–19. doi: 10.47544/johsk.2021.2.4.10

[ref29] Huang Y. (2019). Exploration on the integration of university martial arts teaching and psychological development training. Front. Sport Res. 1, 51–58. doi: 10.25236/FSST.080108

[ref30] Jarman H. K. Marques M. D. McLean S. A. Slater A. Paxton S. J. (2021). Social media, body satisfaction and well-being among adolescents: a mediation model of appearance-ideal internalisation and comparison. Body Image 36, 139–148. doi: 10.1016/j.bodyim.2020.11.005, PMID: 33285385

[ref31] Jiang Z. (2024). Assessment of the health effects of martial arts from the perspective of double difference modeling and its application in modern society. Appl. Math. Nonlinear Sci. 9, 1–14. doi: 10.2478/amns-2024-2299, PMID: 39513035

[ref32] Jianting K. (2025). The relationship between functional motor competence and students' interest in learning Wushu short weapons courses in higher learning institutions in China: coach support as a mediator. Res. J. Psychol. 3, 408–421. doi: 10.59075/rjs.v3i1.80

[ref33] Kang B. Zhao S. Li J. (2024). A111: a study on effect of learning martial arts by different people influenced by Chinese culture. IJPAH 3:111. doi: 10.18122/ijpah.3.3.111.boisestate

[ref34] Kulpinski J. (2018). Teoria wychowania fizycznego. Sandomierz: Wyzsza Szkola Humanistyczno-Przyrodnicza.

[ref35] Leskova I. V. Peredelsky A. A. Zyazin S. Y. Krivoukhov A. A. (2019). Physical culture to strengthen personal and spiritual preferences of Muslims. Teoriya i Praktika Fizicheskoy Kulturythis 8:89.

[ref36] Li H. (2020). Study on the influence of psychological intervention on mood state and coping styles for high-level athletes: a case study for Wushu sport in China. SAGE Open 10, 1–10. doi: 10.1177/2158244020932519, PMID: 40297624

[ref37] Li C. Siriphan C. (2024). The effect of specific training programs to improve agility and flexibility of Wushu Taolu athletes. Int. J. Sociol. Anthropol. Sci. Rev. 4, 23–32. doi: 10.60027/ijsasr.2024.4089

[ref38] Li J. Wang X. Wang L. Kang H. (2022). Effects of artificial intelligence and virtual reality in martial arts sports on students’ physical and mental health. Int. Transact. Electr. Energy Syst. 2022, 1–12. doi: 10.1155/2022/1359243, PMID: 39712885

[ref39] Liu M. Deng H. (2024). Exploring the application and value of martial arts in modern society by combining double difference models. Appl. Math. Nonlinear Sci. 9, 1–13. doi: 10.2478/amns-2024-1551

[ref40] Ma G. (2025). Cell biological mechanisms of muscle fiber type transformation and athletic performance enhancement by martial arts exercise. Mol. Cell. Biomech. 22:979. doi: 10.62617/mcb979

[ref41] Mei B. Yuan L. (2024). Assessing aesthetics, technique, and expression in Wushu performance. J. Educ. Educ. Res. 10, 175–182. doi: 10.54097/ydd0f179

[ref42] Melguizo-Ibáñez E. Zurita-Ortega F. Ubago-Jiménez J. L. López-Gutiérrez C. J. González-Valero G. (2023). An explanatory model of the relationships between sport motivation, anxiety and physical and social self-concept in educational sciences students. Curr. Psychol. 42, 15237–15247. doi: 10.1007/s12144-022-02778-9

[ref43] Monteiro J. R. F. Vecchio F. D. Vasconcelos B. B. Coswig V. S. (2020). Specific Wushu Sanda high-intensity interval training protocol improved physical fitness of amateur athletes': a pilot study. Revista de Artes Marciales Asiáticas 14, 47–55. doi: 10.18002/rama.v14i2.6029

[ref44] Moore B. Dudley D. Woodcock S. (2019). The effects of martial arts participation on mental and psychosocial health outcomes: a randomized controlled trial of a secondary school-based mental health promotion program. BMC Psychol. 7:60. doi: 10.1186/s40359-019-0329-5, PMID: 31511087 PMC6737629

[ref45] Park T. S. (2019). Academic convergence of taekwondo and tourism in Korea. Asia Life Sci. 3, 1085–1092.

[ref46] Partikova V. (2019). Psychological collectivism and mental toughness in traditional Wushu. Doctoral thesis. Hong Kong: Hong Kong Baptist University.

[ref47] Pawelec P. (2020). Martial arts and combat sports. Towards the general theory of fighting arts: book review. Ido Mov. Cult. J. Mart. Arts Anthropol. 20, 54–57. doi: 10.14589/ido.20.1.7

[ref48] Peredelsky A. A. (2016). Two-faced Janus. Sport as a social phenomenon: essence and ontological foundations. monograph (Moscow: Sport).

[ref49] Qian B. (2024). Strategies for informational integration of Wushu teaching and ethnic traditional sports culture. Appl. Math. Nonlinear Sci. 9, 1–18. doi: 10.2478/amns-2024-2835, PMID: 39513035

[ref50] Qiu X. Song X. (2017). Research on Ma Yun Taichi management ideology and practice. Mod. Manag. 7, 493–501. doi: 10.12677/mm.2017.76064

[ref51] Ren Y. (2022). Training fatigue and recovery of Wushu Sanda athletes based on comprehensive environmental testing. Appl. Math. Nonlinear Sci. 8, 845–856. doi: 10.2478/amns.2021.2.00223, PMID: 39513035

[ref52] Rodrigue T. Yong Z. Wen L. (2019). Intercultural communication of Chinese martial arts in Africa. New Media Mass Commun. 84, 63–77. doi: 10.7176/nmmc/84-06

[ref53] Russ G. Bonnema S. J. Erdogan M. F. Durante C. Ngu R. Leenhardt L. (2017). European thyroid association guidelines for ultrasound malignancy risk stratification of thyroid nodules in adults: the EU-TIRADS. Eur. Thyroid J. 6, 225–237. doi: 10.1159/000478927, PMID: 29167761 PMC5652895

[ref54] Russo G. Ottoboni G. (2019). The perceptual-cognitive skills of combat sports athletes: a systematic review. Psychol. Sport Exerc. 44, 60–78. doi: 10.1016/j.psychsport.2019.05.004

[ref55] Santanna E. Li B. (2024). Cultural hybridity and body image formation: exploring the experiences of Wushu male practitioners at the Siberian Chinese martial arts center. Front. Psychol. 15:1435647. doi: 10.3389/fpsyg.2024.1435647, PMID: 39507082 PMC11537934

[ref56] Siswantoyo M. T. Kuswarsantyo K. (2017). Pencak silat dance; developing local genius values in the perspective of tourism business opportunity. Int. J. Appl. Bus. Econ. Res. 15, 639–646.

[ref57] Stephen S. J. Shan G. Banks S. J. Bernick C. Bennett L. L. (2020). The relationship between fighting style, cognition, and regional brain volume in professional combatants: a preliminary examination using brief neurocognitive measures. J. Head Trauma Rehabilit. 35, E280–E287. doi: 10.1097/HTR.0000000000000540, PMID: 31834060

[ref58] Stolyarov V. I. Seiranov S. G. (2021). Theory of physical culture (critical analysis of modern state, technology and results of modernisation): Monograph. Moscow: Moscow State Academy of Physical Culture.

[ref59] Su X. (2016). Reconstruction of tradition: modernity, tourism and shaolin martial arts in the shaolin scenic area, China. Int. J. History Sport 33, 934–950. doi: 10.1080/09523367.2016.1227792

[ref60] Sun Y. Zhang B. Ji A. Sun W. (2022). A study on the impact of Wushu sports health on college students’ mental health. J. Environ. Public Health 2022:5841017. doi: 10.1155/2022/5841017, PMID: 36072490 PMC9444477

[ref61] Sunardi C. (2019). The fighting art of Pencak Silat and its music: from southeast Asian village to global movement ed. by Uwe U. Paetzold and Paul H. Mason. Asian Music 50, 126–129. doi: 10.1353/amu.2019.0007

[ref62] Tao L. (2021). Application of data mining in the analysis of martial arts athlete competition skills and tactics. J. Healthc. Eng. 2021:5574152. doi: 10.1155/2021/5574152, PMID: 33884158 PMC8041537

[ref63] Téllez G. F. Delgado J. E. C. González J. G. Marín J. M. Morales H. N. Rivera M. E. A. M. (2019). Computer aided teaching in the instructional system of martial arts disciplines: a case of application of Triz as a proposal for technological innovation in contact sports. Memorias del Congreso 11, 813–818.

[ref64] Wang Z. (2024). The influence of Chinese martial arts teaching on the moral quality and achievement motivation of college students in China. J. Educ. Educ. Res. 8, 241–250. doi: 10.54097/wpeysw72

[ref65] Wang L. Feng C. (2023). Establishment and application of traditional Wushu intelligent learning resource database under the background of big data. Appl. Math. Nonlinear Sci. 8, 1369–1386. doi: 10.2478/amns.2023.1.00041

[ref66] Wang Q. Salleh M. (2024). Exploring the influence of student knowledge, interests, and values on educational outcomes in Wushu education: a conceptual framework. Int. J. Acad. Res. Progress. Educ. Dev. 13, 1257–1263. doi: 10.6007/ijarped/v13-i3/21891, PMID: 39300133

[ref67] Wu Z. Huang Z. Zhang Y. (2020). Discussion on the history and genres of traditional Wushu. DEStech Trans. Soc. Sci. Educ. Human Sci. 2020:34462. doi: 10.12783/dtssehs/icesd2020/34462

[ref68] Yang X. Hung K. Huang W. J. Tseng Y. P. (2019). Tourism representation by DMOs at religious sites: a case of shaolin Temple, China. Tour. Manag. 75, 569–581. doi: 10.1016/j.tourman.2019.06.017

[ref69] Ying L. (2024). Optimizing performance in Wushu Sanda: the combined influence of mental preparation and physical training on competition success. Int. J. Educ. Human. 16, 242–246. doi: 10.54097/p73ew548

[ref70] Yu X. (2020). Fatigue and recovery of Wushu athletes based on fatigue damage model. IOP Conf. Ser. Mater. Sci. Eng. 914:012016. doi: 10.1088/1757-899X/914/1/012016

[ref71] Yu P. Yang X. Guo Q. Guan J. Chen G. (2025). Spatial distribution characteristics and influencing factors of China martial arts schools based on Baidu map API. PLoS One 20:e0314588. doi: 10.1371/journal.pone.0314588, PMID: 40036209 PMC11878914

[ref72] Zeng Q. (2022). Research on the explosive training skills of Wushu Sanda athletes. IJSEA 11, 142–144. doi: 10.7753/ijsea1110.1006

[ref73] Zhang J. (2021). Research on the application and development of multimedia teaching in college Wushu course based on internet technology. J. Phys. Conf. Ser. 1915:022075. doi: 10.1088/1742-6596/1915/2/022075

[ref74] Zhang Y. Chin J. Reekie S. (2019). Education in the Chinese national sport system: experiences of professional Wushu athletes. Sport Soc. 22, 1466–1480. doi: 10.1080/17430437.2018.1529168

[ref75] Zhang Z. Hongsaenyatham P. Siriphan C. (2024). The construction of Wushu teaching strategy in higher education based on WSR theory. IJSASR 4, 163–174. doi: 10.60027/ijsasr.2024.4000

[ref76] Zhang J. Qu Q. An M. Li M. Li K. Kim S. (2022). Influence of sports biomechanics on martial arts sports and comprehensive neuromuscular control under the background of artificial intelligence. Contrast Media Molec. Imag. 2022:9228838. doi: 10.1155/2022/9228838, PMID: 36003995 PMC9385289

[ref77] Zhou H. (2024). Research on the intrinsic mechanism and educational philosophy of “Kecheng-Sizheng” for Wushu. J. Educ. Teach. 2, 111–117. doi: 10.59825/jet.2024.2.3.111

[ref78] Zilai Q. Lin W. (2018). Barriers to intercultural communication of martial arts. J. Phys. Educ. 4, 91–93.

