# Supplementary material for: Traditional Chinese martial art Wushu to improve the mental state and physical fitness of students: designing space for optimal practice and training
Source: Front Psychol. 2025 Jun 18;16:1581226. doi: 10.3389/fpsyg.2025.1581226 (PMC12213914; doi:10.3389/fpsyg.2025.1581226)
Supplement: Supplementary file 1 [file Supplementary_file_1.docx]

Appendix 1

Sample questions for assessing wushu training grounds

| Category | Question | Response type | Assignment | Attitudes towards the design of the space | Scientific basis |
| --- | --- | --- | --- | --- | --- |
| Acoustics | How does background noise affect focusing? | Likert scale | Assesses auditory distractions | Determines the need for soundproofing | Environmental psychology |
| Illumination | Is the lighting uniform and glare-free? | Yes/No | Assessment of visibility and eye strain | Optimises light diffusion | Ergonomic design |
| Floor coverings | Does the surface effectively absorb shock? | Likert scale | Measures to prevent injuries | Determines the choice of material | Biomechanics |
| Temperature | Does temperature affect endurance levels? | Open | Examines thermoregulation in the learning process | Informs about climate control settings | Sports physiology |
| Ventilation | Is air quality conducive to high-intensity exercise? | Likert scale | Assesses oxygen availability | Adjustment of air supply systems | Exercise science |
| Ceiling height | Does the ceiling height allow for unobstructed movement? | Yes/No | Provides freedom for airborne technology | Influences vertical space planning | Kinesiology |
| Wall mirrors | How does mirror placement affect self-correction? | Likert scale | Determines dependence on visual feedback | Optimal arrangement of reflective surfaces | Motor learning |
| Study areas | Are there separate areas for different types of learning? | Yes/No | Prevents overpopulation | Organises the spatial distribution | Facilities management |
| Equipment storage | Is it convenient to store the equipment? | Yes/No | Reduces transition time | Designing spatial ergonomics | Industrial design |
| Instructor positioning | How does instructor placement affect engagement? | Likert scale | Determines the visibility of the instructor | Adjusts the layout of the room | Pedagogical design |
| Cognitive load | How does the organisation of space affect concentration? | Open | Examines mental distractions | Aligning spatial structuring with cognitive flow | Psychology of education |
| Colour range | Does the choice of colour affect the intensity of your workout? | Likert scale | Assesses the psychological impact | Determines the aesthetic-functional balance | Colour theory |
| Utilisation of technology | How do digital tools affect feedback in training? | Multiple choice | Evaluates dependence on technological tools | Strategic integration of screens/sensors | Human-computer interaction |
| Study stream | Does the layout impede traffic? | Yes/No | Identifies spatial inefficiencies | Optimises traffic flow | Spatial planning |
| Levels of distraction | How do external stimuli affect concentration? | Open | Identifies sources of cognitive overload | Minimising unnecessary external inputs | Cognitive ergonomics |
| Perception of fatigue | Does room layout affect perceived fatigue? | Likert scale | Assesses mental toughness | Modification of the layout to optimise traffic | Psychophysiology |
| Cultural symbolism | Does the environment reflect the cultural heritage of wushu? | Yes/No | Evaluates the psychological connection to practice | Includes cultural aesthetics | Cultural studies |
| Spatial flexibility | Can the space be adapted for different learning needs? | Likert scale | Defines the versatility of the design | Creating modular configurations | Architectural flexibility |

Source: author's elaboration

Appendix 2

Functional and spatial segmentation of wushu training environment

| Zone | Spatial configuration | Main function | Psychological impact | Kinetic considerations | Symbolic/philosophical embedding |
| --- | --- | --- | --- | --- | --- |
| Meditation hall | Round, closed, with dim lighting | Cognitive centring, concentration training | Causes autonomic regulation, increases inner awareness | Requires smoothness in changing postures, controlled synchronisation of breathing | Embodies “静” (Jìng), the principle of immobility as potential |
| Kata / Forms | Open, high ceiling, adjustable airflow | Precision in taolu, biomechanics refinement | Strengthens procedural memory, consolidation of motor schemas | Requires symmetry in spatial design, adherence to spatial grid lines | Reflects the “形” (Xíng)-form as the outward embodiment of the intention |
| Weapons section | Linear structure, distinct boundary markers | Weapons manipulation, range calibration | Enhances proprioceptive differentiation, improves fine motor control | Requires spatial subdivision for short and long distance applications | Means “力” (Lì) – force as a controlled projection of internal energy |
| Combat Zone / Separation Zone | Semi-enclosed, with variable floor texture | Live Combat Training, tactical adjustments | Increases adaptability to situation, instils stress resistance | Includes training rapid deceleration, variable movement initiation. | Represents “劲” (Jìn)-force as an adaptive response to external stimuli. |
| Group synchronisation space | Expanding mirror walls, optimising acoustics | Collective exercises, rhythm regulation | Develops spatial perception, collective kinaesthetic learning | Promotes multi-directional interaction, engaging movement | Symbolises “和” (Hé)-harmony as spatial and temporal coherence |
| Recovery and reflection zone | Ergonomically designed seats with baffles and temperature control | Post-training analysis, controlled decompression | Enhances experience consolidation, psychological resetting | Physiological adaptation required, multisensory decompression | Embodies “悟” (Wù)-penetration as a function of reflection |

Source: author's elaboration
